# A Review of the Properties and CVD Synthesis of Coiled Carbon Nanotubes

**DOI:** 10.3390/ma3042618

**Published:** 2010-04-12

**Authors:** Dóra Fejes, Klára Hernádi

**Affiliations:** Department of Applied and Environmental Chemistry, University of Szeged, Rerrich B. tér 1., Szeged, H-6720, Hungary; E-Mail: fejesd@chem.u-szeged.hu (D.F.)

**Keywords:** coiled carbon nanotubes, CVD method, electron microscopy

## Abstract

The CVD route for carbon nanotube production has become a popular method to make large amounts of multiwall carbon nanotubes. The structure, morphology and size of carbon materials depend critically on the catalyst preparation and deposition conditions. According to current knowledge, CVD method is the only process which can produce carbon nanocoils. These nanocoils are perfect candidates for nanotechnology applications. One might indeed hope that these coils would have the extraordinary stiffness displayed by straight nanotubes. Based on theoretical studies, regular coiled nanotubes exhibit exceptional mechanical, electrical, and magnetic properties due to the combination of their peculiar helical morphology and the fascinating properties of nanotubes. In spite of its technological interest, relatively low attention has been paid to this special field. In this paper we attempt to summarize results obtained until now.

## 1. Introduction

Carbon is an enigmatical element which has numerous allotropic forms whose structures can be tailored to different kinds of conformations and morphologies. Besides well-known diamond and graphite various materials such as fullerenes (buckyballs) [1], carbon nanotubes [2], and (nano)fibers [3], (nano)rods [4], onions [5], foams [6], buds [7], *etc.* can be fabricated in the micrometer to nanometer scales. Among these micro- and nanostructures, coiled carbon nanotubes have very interesting morphology and many unique characteristics. Due to their good chiral conductivity [8,9], large surface area [8], super-elasticity [10], they possess several new potencial applications [11].

Since the discovery of carbon nanotubes, their applications have benefitted a wide range of engineering, applied physics and biomaterials areas, because of their superior mechanical and electrical properties. Carbon nanotubes have played a fundamental role in leading the extensive development of nanoscience and nanotechnology in both scientific research and industrial innovations. The extent of this interest is evident by the fact that carbon nanotubes are the subject of study of about seven research papers each day, excluding book chapters and reviews [12]. In a first approach the geometrical description of carbon nanotubes is very simple: a cylinder of a rolled graphene sheet (single-walled nanotubes: SWNTs) or multiple concentric cylinders consisting of rolled graphene sheets (multi-walled nanotubes: MWNTs) [13,14].

**Figure 1:**
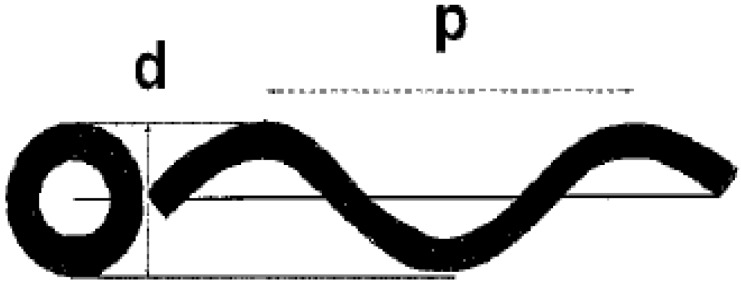
Coiled tube with its projection (left) showing *d*) helix diameter and *p*) coil pitch. Reproduced from [25] with permission.

The existence of helically coiled carbon nanotubes was first predicted by Ihara *et al*. and Dunlap in the early nineties [15,16,17,18] and a few years later a Belgian research group reported their experimental observation [19]. For the characterizations of the geometry of coiled carbon nanotubes helix diameter and coil pitch (the distance between adjacent corresponding points along the axis of the helix) are commonly used (Figure 1) [25]. Carbon nanotube with regular coiled structure can be generated with periodic incorporation of pentagon and heptagon pairs into a hexagonal carbon framework *via* creating positively and negatively curved surfaces [20]. Since the insertion of pentagons and heptagons into hexagons induces significant changes in the electronic structures [21,22], helical and toroidal forms possess remarkable electrical and magnetic properties, which are rather different than that of cylindrical tubes [23,30]. Using computer simulation with molecular dynamics calculations it was proved that coiled carbon nanotubes are both thermodynamically and energetically stable [24]. Consequently, the coiled carbon nanotubes are expected to show exceptional properties and to afford various applications in the near future. In spite of its technological interest, relatively low theoretical and experimental attention was paid to this fascinating carbon material [12].

For the synthesis of carbon nanotubes, several methods have been reported. In the early ninties the arc-discharge method developed for C_60_ synthesis supplied a very surprising result, namely the growth of fullerene tubes was observed on the carbon cathode. In this method carbon nanotubes are always grown out of the ends of carbon electrodes of the arc but they have never been found in the soot condensing from the vapour. Maybe this recognition led to the discovery of other nanotube synthesis methods such as plasma decomposition of hydrocarbons and co-evaporating catalyst during a carbon arc-discharge. The CVD route for nanotube production has become a popular method to make large amounts of multiwall carbon nanotubes. Compared with other synthesis methods, the selectivity of this process to carbon nanotubes is significantly higher and, consequently the final product contains much less amorphous carbon. It is indeed the best way of producing large quantities for materials science applications, such as the fabrication of nanotube-reinforced composites. Occurrence of coil-shaped fibers in the jungle of straight catalytically grown nanotubes has been noticed from the early days of studying their catalytic synthesis. These coiled tubes do not appear when an arc-discharge process is used, nor any other process. [25].

The morphologies of coiled carbon nanotubes and carbon microcoils are rather different, however, similar features of their synthesis are indisputable and prominent selectivity obtained by Motojima *et al*. is remarkable. A few decades ago, the growth of these helical carbon microcoils was basically accidental, and both reproducibility and the yield were very low. Recently significal improvement occurred on this field, a large-scale preparation technique for regularly coiled carbon microfibers of micrometer diameter was developed. The method having high reproducibility and high coil yield produces carbon coils with circular fiber cross-section and extreme elasticity (10-15 times higher) [26,27,28]. The name ‘‘super-elastic carbon coils’’ originates from the same group [10].

Regularly coiled carbon nanotubes, their synthesis, their structure, formation mechanism and theoretical aspects are mysterious points. In this review we give an up-to-date summary of scientific results accumulated until now on this field. Preliminary observations and their evaluation might generate new ideas and further motivation for relevant scientific community to clarify fascinating questions.

## 2. Theoretical Calculations and Properties of Carbon Nanotubes

**Figure 2:**
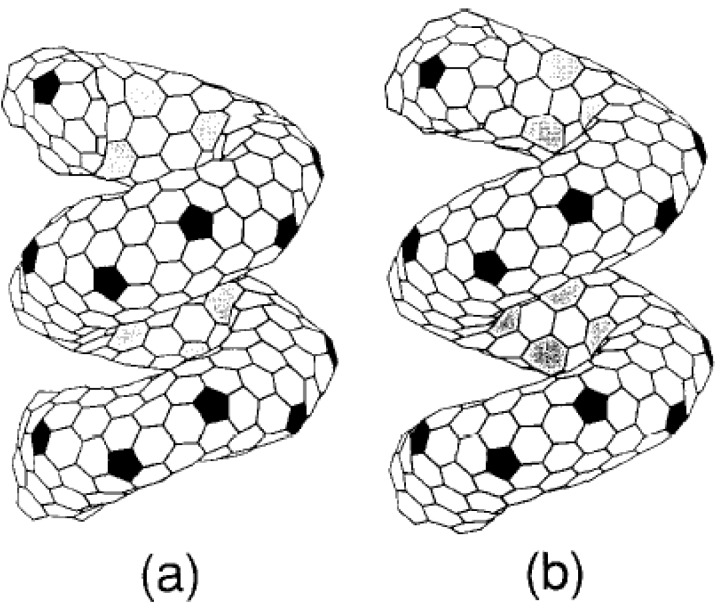
Helically coiled form C_360_: one pitch contains a torus C_360_. (a) coil length = 12.9 Å and, (b) coil length = 13.23 Å. The tiling pattern of heptagons in the inner ridge line is changed, though the pattern of pentagons in the outer ridge line remains upon changing the coil length. Reproduced from [30] with permission.

In the early study of the growth of carbon nanotubes, Iijima reported that incorporation of seven-fold rings of carbon atoms (heptagons) into a hexagonal sheet results in a negatively curved surface. Theoretically, a crystal with only negatively curved surface, a so-called minimal surface can be thus constructed [29]. The positively curved surface can be created by the insertion of pentagons (five-fold rings of carbon atoms), and a negatively curved surface can be created by heptagons. With the combination of these surfaces, namely tiling the surface with pentagons, heptagons, and hexagons, in principle, a unique form of carbon having novel features and potential considerable technological interest can be constructed (Figure 2) [30].

The presence of a large amount of curved and coiled nanotubes among the tubes produced by the catalytic method stimulated several studies on the theoretical aspect of the coiling mechanism [19,20,31,32,33,34]. Based on observations from high-resolution electron microscopy and electron diffraction, it was proposed that the curving and coiling could be accomplished by the occurrence of ‘knees’, *i.e.*, two straight cylindrical tube sections of the same diameter connecting at an angle.

Such knees can be obtained via the insertion in the plane of the knee of diametrically opposed pentagonal and heptagonal carbon rings in the hexagonal networks. The heptagon is on the inner side of the knee and the pentagon is on the outer side. The possibility of such construction was predicted by Dunlap [18,35,36]. Theoretical models of curved nanotubes forming tori of irregular diameters have also been described by Itoh and Ihara [17,37].

Based on energetic and thermodynamic stability considerations, the above-mentioned authors reported the possible existence of various forms of helically coiled and toroidal structures. It was also highlighted that the variety of patterns in the outer and inner surface of these structures indicates that many other stable cage carbon structures may exist [15,16,17,18,38,39]. Itoh and Ihara also proved that the molecules in a one dimensional chain, or a two-dimensional plane, or a three-dimensional supermolecule are possible extended structures of tori with rich applications. In the helically coiled form, coils may be able to transform into other forms. They highlighted that it would be interesting if the proposed structure and its variant forms-combinations of helix with toroidal forms (helical coiling around the tube, nested helical forms, coiled structure of higher order such as supercoil observed in biological systems) could be constructed in a controlled manner from the graphitic carbon cage. Interesting electrical and magnetic properties of helical and toroidal srtucture which are defenitely different than those observed in cylindrical carbon nanotubes were also predicted [30].

Su *et al*. studied the shape formation process of carbon nanotubes and described a string equation for the possible existing shapes of the axis curve of multiwalled carbon nanotubes (MWNTs). It was shown that there is a threshold condition for the formation of straight MWNTs; below that value straight MWNTs became unstable and a shape deformation would occur. In particular, the optimal ratio of pitch *p* and radius *r_0_* for such a coil was found to be equal to 2π, which was in good agreement with the recent experiment observations obtained by Zhang *et al*. [19,24].

**Figure 3:**
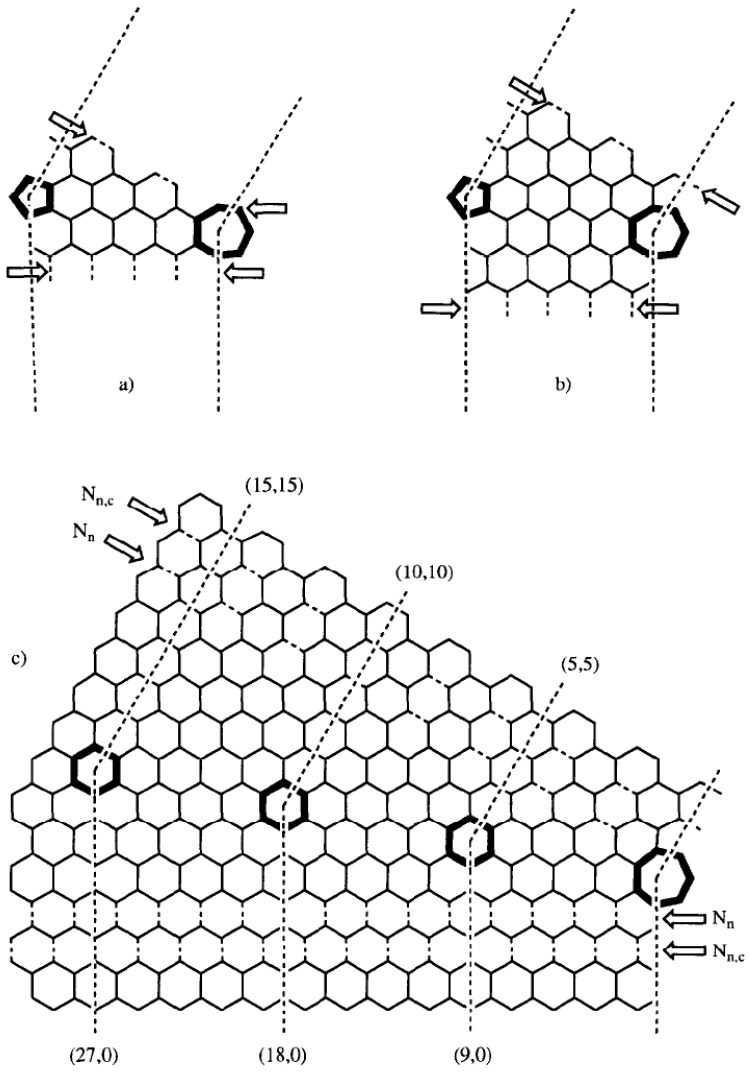
Planar presentation of the (9n,0)-(5n,5n) knees, having a 36° bend angle produced by a heptagon–pentagon pair on the equatorial plane. The arrows show the dotted line of bonds where the knee *N_n_* or *N_n,c_* is connected to the corresponding straight tubules: (a) knee *N_n_* for n = 1; (b) stretched knee *N_n,c_* for n = 1 and c = 38; (c) general knees *N_n_* and *N_n,c_*. Reproduced from [33] with permission.

Fonseca and co-workers elaborated models of perfect tubule connections leading to curved nanotubes, tori or coils using the heptagon-pentagon construction of Dunlap [18,35]. In order to understand the mechanisms of formation of perfectly graphitized multilayered nanotubes, models of concentric tubules at distances close to the characteristic graphite distance and with various types of knee were built (Figure 3).

Perfectly graphitizable (chiral and achiral) knees are described, and relations are established between the tubules and their graphitic layers. A growth mechanism at a molecular level was described by the same authors in order to explain the formation of knees, consequently other derivates such as tori and coils [33,37].

On the theoretical side, carbon toroids can be considered [40], either by introducing curvature inducing defects as in the helical nanotubes, or by elastically bending a straight nanotube so that it closes upon itself. In the latter case, the lower limit for the diameter is found to be about 200 nm [40]. Ahlskog *et al*. report on the observation by AFM and SEM of ring formations in catalytically grown carbon nanotube material. The rings have the same dimensions (diameter and thickness) as was observed in laser grown SWNT material [41], which may suggest a common origin. Possible links to the observed helical nanotubes in the same catalytically grown material [42] were discussed. Their observations contrast with previous reports of the nanotube rings as genuine toroids. Still, since the tools used in that work are not capable of definitely resolving whether the rings are toroids or small coils, they conclude that more elaborate investigations are needed to address this question. In any case, the rings are interesting as objects for various future experiments to study their mechanical and electronic properties [43].

Lambin and his co-workers solved problems of the first models which were based on the very regular incorporation of a small fraction of pentagons and heptagons into a perfect hexagonal lattice. The authors revealed difficulties of this mechanism in which such a regular incorporation of isolated non-hexagonal rings takes place. Instead, they offered a new family of Haeckelite nanotubes which was generated in a systematic way by rolling up a two dimensional three-fold coordinated carbon network composed of pentagon–heptagon pairs and hexagons in a 2:3 proportion. In their model, the non-hexagonal rings were treated as normal building blocks of the structure. They showed that in a similar way like straight-carbon nanotubes were wrapped from a graphene sheet, it was possible to wrap systematically tubular-carbon nanostructures which were regularly coiled and in which the non-hexagonal rings did not constitute defects, but were regular building blocks of the structure. *Via* cohesion energy calculations it was showed that the stability of the generated three-dimensional Haeckelite structures was between that of straight carbon nanotubes and that of C_60_. Electronic density of states of the Haeckelite computed with a tightbinding Hamiltonian that includes the orbitals only showed that these structures are semiconducting. The comparison of Haeckelite structures and experimental findings was also discussed. During experimental observations they proved that Haeckelite type structures could be produced by other growth procedures developed for carbon nanotube synthesis. This work significantly helped the optimization of the growth conditions resulting in a selective production of Haeckelite structures [44].

Biró *et al*. observed carbon nanotube “knees”, Y-branched and coiled single wall carbon nanotubes grown on a graphite substrate by the decomposition of fullerene either at room temperature or at 450 °C in the presence of transition metal (Ni particles of 200 nm size). The characterization of as-prepared material was carried out by direct STM measurements. The formation of the carbon nanostructures with non-hexagonal rings is ascribed to both the templating effect of HOPG and the growth at room temperature. The observed coiled carbon nanotubes were tentatively identified with the theoretically predicted haeckelite [45,46].

For the experimental determination of the mechanical properties of coiled carbon nanotubes, in particular, the Young’s modulus (*E*), the shear modulus (*G*) and Poisson’s ratio (*υ*), a few methods were reported recently [47,48]. Since *E* = 2(1+*υ*)*G*, knowing two parameters is sufficient. Measuring uniaxial tension/compression and bending are typical methods for characterization of carbon nanotubes [49]. The simplest way to characterize cabon nanocoils is uniaxial stretching. For e.g. using a nanomanipulator, a carbon nanocoil was extended to a maximum elongation of 33% with the relationship of elongation (*δ*) *versus* tension load along the coil axis (*P*) recorded [47,48]. But for a long time no comparison with the real *δ–P* curve of coiled nanotubes over a long stretching distance has been reported. To supplement the mechanics of coiled nanotubes in uniaxial tension was reported by Huang. In his work the estimations for double coils were obtained. He observed a good agreement in the whole stretching process (about 30% elongation) in comparison with the experimental result. It was found that as an alternative to, *i.e.,* the inter-atomic potential method, given the initial geometry, the shear modulus of the tube can be determined by the uniaxial tensile test. However, the analysis also revealed that it was difficult to determine the Poisson ratio precisely [50].

Multi-walled coiled nanotubes with different coil diameter and coil pitch showed no significant differences from the normal multi-walled nanotubes in their Raman spectra and X-ray diffraction patterna [51]. The Raman spectra indicated that the structures of all these carbon coils were nanocrystalline phases in amorphous networks in spite of the different catalysts and preparation conditions [52].

## 3. Synthesis of Carbon Microcoils

The growth mechanisms of catalytic carbon coils synthesis by chemical vapor deposition (CVD) are of great interest in the research of helical/spiral materials. In the past decades, several growth models were published to clarify the formations of carbon coils with unique shapes and morphologies [53]. A number of excellent papers and reviews about the properties, morphology, growth mechanisms, preparations, and applications of carbon coils appeared by Rodriguez [54], De Jong and Geus [55], and Motojima and Chen [53]. While the CVD production and characterizations of carbon microcoils were substantially investigated since their early studies [56,57], the electric transport properties of these coiled carbon structures were only poorly known. For e.g., the electronic properties of carbon microcoils were studied exclusively by Motojima *et al*. [53]. The electric conductivity of such a microcoil having the diameter of several micrometers and the length of several millimeters was found 30-100 S/cm, where the temperature dependence (~400-10 K) of conductivity indicated both semi-conductive behavior with an activation energy of 4 meV and a variable range hopping transport mechanism in the carbon microcoil [58,11].

Motojima *et al*. obtained carbon microcoils by the Ni catalyzed pyrolysis of acetylene at 770 °C using thiophene as an impurity with and without an external electromagnetic (EM) field and by applying or not applying a bias voltage to the substrate. They examined the effect of the external EM field and bias voltage applied to the substate on the vapor growth, morphology and properties of the carbon coils. The electromagnetic field applied to the reaction tube and the bias voltage applied to the substrate significantly affected not only the growth of carbon coils, but also their morphology and properties. The highest coil yield of 30–35 mg/cm^2^ of substrate was obtained with an external EM field and with a DC or AC bias of 600–1000 V, and this value was 1.9–2.0 times higher than that obtained without an EM field and bias voltage. The carbon coils obtained with the EM field were very regularly coiled flat coils with a flat or long diameter of 5.5–6.0 mm and a coil pitch of 2.5 mm irrespective of the application of the DC or AC bias. On the other hand, the carbon coils obtained without the EM field had a circular or elliptical fiber cross section, a coil diameter of 4.5–5.0 mm and a very small coil pitch of 0.4–0.5 mm. The density of the carbon coils obtained with the external EM field and bias voltage was 1.79–1.84 g/cm^3^. These values were higher than the value of 1.72–1.74 g/cm^3^ obtained without the EM field and bias voltage [59].

Carbon microcoils of various shapes were obtained by the nickel catalyzed pyrolysis of acetylene. There were two kinds of carbon microcoils based on the shape of the cross section of the carbon fibers that compose the carbon coils: circular carbon coils with circular fiber cross sections and flat carbon coils with slender form fiber cross section. It was considered that the flat coil obtained by a long reaction time is formed by the change in the shape of the catalyst grain from a cubicform to a slender form in an electromagnetic field [60].

According to transmission electron microscopy observations, carbon deposits obtained in the decomposition of acetylene over Fe_3_C(+SnCl_2_) may also contain coiled shape carbon nanofibers. In the presence of SnO-doped Fe_3_C, carbon deposit contained mixed quality carbon fibers having smaller as well as larger diameters. It is interesting observation that pure Fe_3_C was found to absolutely inactive in acetylene decomposition, no significant amount of carbon could be detected on its surface after CVD reaction [61].

Protein-like single-helix carbon microcoils were prepared in a Ni-alloy catalyzed CVD process. The product was embedded into polysilicone matrix in order to form artificial skin biomimetic tactile sensor elements. Changes in electrical parameters under the applied loads were investigated, with a comparison with double-helix carbon microcoils. It was found that the single-helix carbon microcoil sensor elements were more stable and sensitive than that of the latter sensor elements [62].

**Figure 4:**
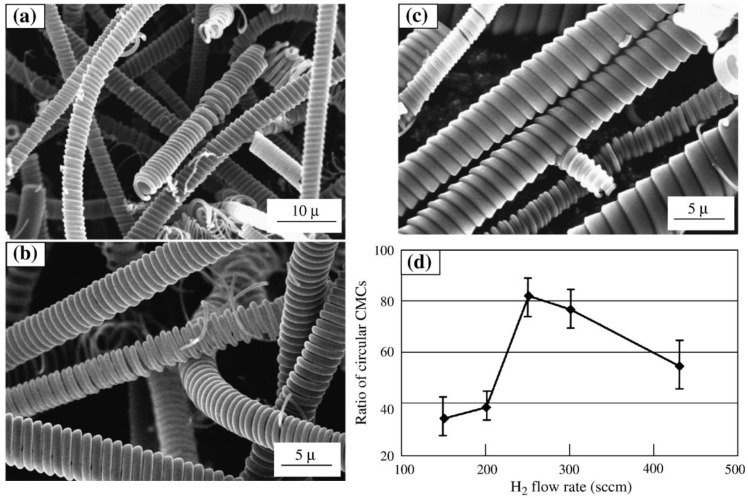
(a–c) SEM images of the CMCs obtained with different H_2_ flow rates when N_2_ was also introduced. H_2_ flow rate: (a) H_2_ 150 sccm, (b) H_2_ 250 sccm, and (c) H_2_ 435 sccm. Other gas flow rates: C_2_H_2_ = 83 sccm, N_2_ = 400 sccm, H_2_S/H_2_ = 70 sccm. Reaction temperature: 770 °C; reaction time: 60 min. (d) A summary of the influence of the H_2_ flow rates. Reproduced from [63] with permission.

Another interesting paper was published about the influence of CVD conditions on the growth of carbon microcoils by Motojima *et al*. They have proposed that the shape of the fiber cross-section was usually determined by the shape of the catalyst grain; fibers with a circular cross-section are generally formed from a cubic or a coaxial-shaped catalyst grain, and fibers with rectangular or flat cross-section were formed from a rectangular or flat-shaped one [60]. It was found that the ratio of the circular carbon microcoils in the deposits could be controlled by adjusting the flow rate of H_2_ without the addition of inert gases and by varying the growth time. Furthermore, the carbon microcoils obtained by the addition of H_2_O vapor showed a more regular coiling pattern and a higher elasticity than those obtained without H_2_O. It can be seen in Figure 4 that when the H_2_ flow rate was 250 sccm, the content of the circular carbon microcoils in the deposits was the highest: 85-100% [63].

Using a symmetric growth model, helical carbon nanofibers of high yield were synthesized by the catalytic decomposition of acetylene at 241 °C with Cu–Ni alloy nanoparticles which were prepared by the hydrogen-arc plasma method. The morphology and microstructure of the coiled carbon nanofibers was determined in detail. The two helical fibers had opposite helicity, but had identical cycle number, coil diameter, coil length and fiber diameter [64].

Three-dimensional 3D spring-like carbon nanocoils were obtained by the catalytic pyrolysis of acetylene using an iron-containing catalyst. Using Fe-rich catalyst systems at the reaction temperature 750–790 °C, the spring-like carbon nanocoils were obtained with nearly 100% purity. The catalyst particles were present on the growth tips, and tip morphology showed that the coiled nanofibers grew by mono-directional growth mode. According to characterization data the spring-like carbon nanocoils were considered to be composed basically of two coils fused each others [65].

Single-helical carbon nanocoils with a coil diameter of 300–400 nm was prepared using Fe-based alloy catalysts at 700–800 °C with 60% yield and 100% purity. The catalyst particle observed on the growth tip was identified as Fe_5_C_2_ or Fe_7_C_3_ single crystal, in which some of the Fe atoms were substituted by Cr atoms. Carbon nanoparticles with a helical structure were observed on the surface of the catalyst, and it was supposed that the carbon nanoparticles have the potential to be helical carbon supply sources. Namely, the catalyst face supplied microscopic carbon nanoparticles by helical deposition patterns on the catalyst surface to form macroscopic helical patterns of carbon nanocoils. Another interesting finding was that graphitic capsule-like carbon layers composed of 5–10 layers formed after heat treatment at 3000 °C in an inert gas for 6 hours [66].

## 4. Synthesis of Coiled Carbon Nanotubes

**Figure 5:**
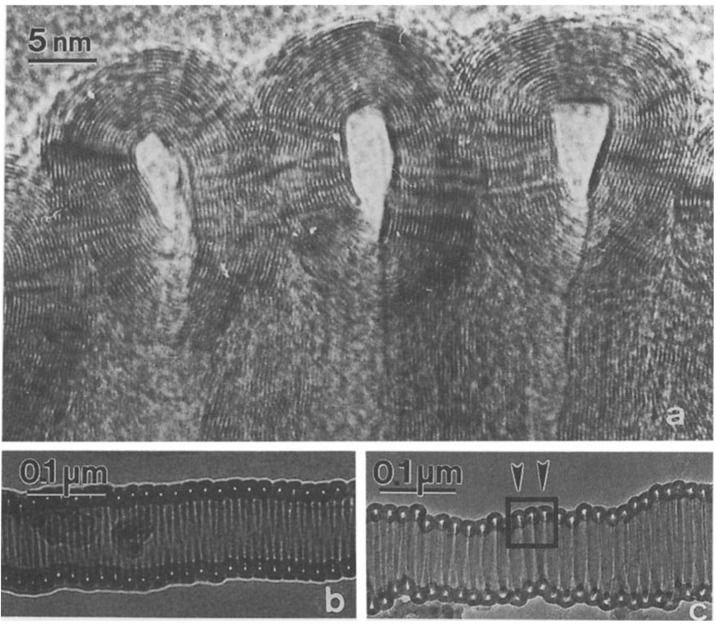
HREM image of a tightly wound helix-shaped carbon nanotubule; insets show two such tubules at lower magnification. Reproduced from [67] with permission.

The first experimental observation for the production of coiled carbon nanotubes was in 1994, when Zhang *et al*. in a Belgian research group observed the multi-walled coiled carbon nanotubes with inner and outer diameter of 15-20 nm in the sample grown by catalytic decomposition of acetylene over silica-supported Co catalyst at 700 °C. The catalytic decomposition of acetylene was carried out in a flow reactor at atmospheric pressure. It was suggested that the coiled tubules consist of regularly polygonized helices where the bends are related to pairs of pentagon-heptagon carbon rings in the hexagonal network [67]. One of the very first coiled carbon nanotubes can be seen in Figure 5.

**Figure 6:**
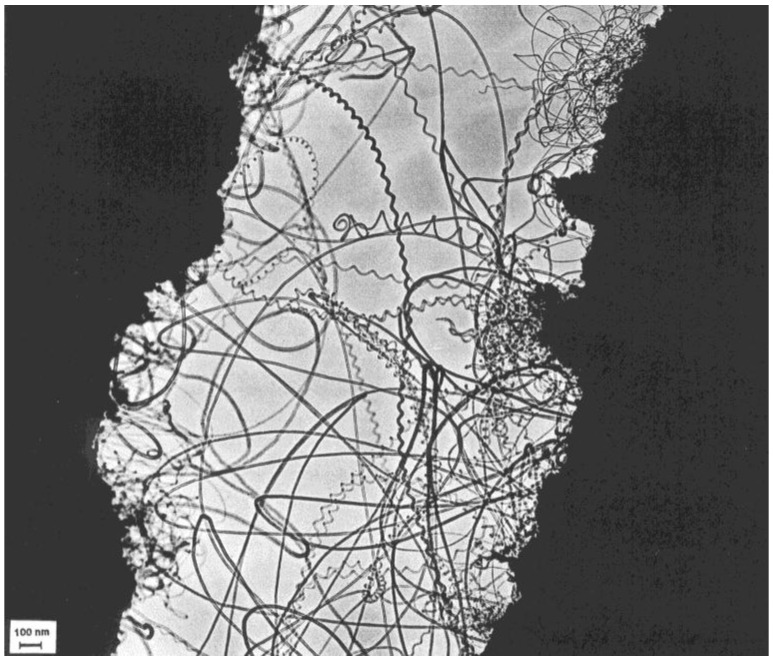
TEM image of helical carbon nanotubes formed in the decomposition of acetylene at 700 °C over Co/silica prepared by method A (pH = 9). Reproduced from [68] with permission.

For the catalyst prepared from Co-acetate solution of pH = 9, the quality of carbon nanotubes in the deposit after CVD has also been found to be very good. The only difference observed by electron microscopy was the definitely higher amount of coiled structures, as illustrated in Figure 6. While in the case of samples pH 8 and 9 every catalyst particle was covered by regular carbon nanotubes after the reaction, the composition of the product obtained over the catalyst pH lower than 7 was more heterogeneous. Much fewer particles were covered by well-turbostratic tubes, while the relative amount of irregular tubes and fibers increased considerably [68].

Bai reported a method to prepare spiral carbon nanotubes with controlled diameter using catalytic decomposition of acetylene on alumina support. Coiled carbon structure growth was carried out through catalytic decomposition of acetylene at 650 °C and at slightly higher than atmosphere pressure. A porous alumina layer from aluminium surface anodisation was used as substrate. The diameter nanotube coils ranged from a few nanometers to a few micrometers, depending mainly on the metal catalysts size. Through the careful choice of alumina pore size and electrochemical metal deposition conditions, he was able to produce the coiled carbon structure with more or less controlled morphology. DC electrochemical deposition with iron sulphate solution was found to be the most favourable condition catalyst preparation which produced large quantity of coiled carbon structure [69].

Hou *et al*. produced helically shaped multiwalled carbon nanotubes (HCNTs) with the diameter of 30-80 nm by co-pyrolysis of Fe(CO)_5_ as floating catalyst precursor and pyridine or toluene as carbon source at a temperature of 1,050-1,150 °C under flow of hydrogen. At lower temperatures they found only carbon-coated iron nanoparticles. Concerning the growths mechanism of coiled structures they found that the metal nanoparticles were in various shapes at the tips of HCNTs, which led to the assumption that tube growth could occur by the so-called tip model. The worm, taper or droplet-curved shape of the nanoparticles at the ends of HCNTs suggested that the metal nanoparticle might be in its molten state at higher temperatures [70].

Carbon nanotube material produced by catalytic decomposition of carbon sources is predominantly multiwalled, with only rare observations of SWNTs [71]. An average helical nanotube is described by the coil diameter, where observations range from 10 nm to 1 mm, and the pitch (the distance between adjacent corresponding points along the axis of the helix), which has been observed to take values from 10 nm up to 5 mm. Careful TEM analysis of the helical nanotubes [19] suggests that these nanotubes are mostly polygonized, consisting of straight segments, and the turning being due to the appearance of pentagon–heptagon pairs in the hexagonal network forming the wall of a perfect straight carbon nanotube. Each defect pair would twist the nanotube about an angle α (with 0°<α<36°), so that at least 10 pairs would be needed to cause one turn [43].

A. Szabó and her coworkers analyzed the shape of more than 300 coiled carbon nanotubes. The statistical, HRTEM and continuum elasticity investigations concordantly showed that the coiling of carbon nanotubes had structural origin while various external factors probably created the specific conditions which made that the coiled shape was more favorable than the straight one. On the basis of the analysis it could be concluded that the most frequently found coiled carbon nanotubes were grouped in certain stability ‘‘islands’’ in their geometrical configuration space, which were reproduced in different experiments and different laboratories. It was interpreted as indirect evidence that the way in which carbon nanotubes were coiled had an intrinsic, structural origin and it was not decided by external factors. The possible role of impurities like nitrogen and sulfur in promoting the production of coiled carbon nanotubes was also pointed out in that work [72].

Coiled carbon nanotubes were prepared by CVD on traditional silica-supported Co nanoparticles under reduced pressure and at lower gas flow rates. The selected area electron diffraction patterns suggested that the helix is polygonized, which is further confirmed by HRTEM. On the basis of the heptagon-pentagon construction theory, they proposed the helix formation mechanism which involved a carbon core formation on a catalytic particle followed by carbon helices growth controlled by kinetics [73].

Liu *et al*. produced spiral carbon nanofibers which were synthesized using the AlPO_4_-5 supported Pd_2_(dba)_3_ catalyst prepared by the impregnation method. It was noted that the synthesis of spiral carbon nanofibers was very sensitive to the reaction temperature. Spiral nanostructures were observed only within a narrow temperature range around 700 °C. The transport behavior indicated the sample consisting of spiral nanofibers having different pitches and radius can be explained in the scenario of the heterogeneous model. [74].

**Figure 7:**
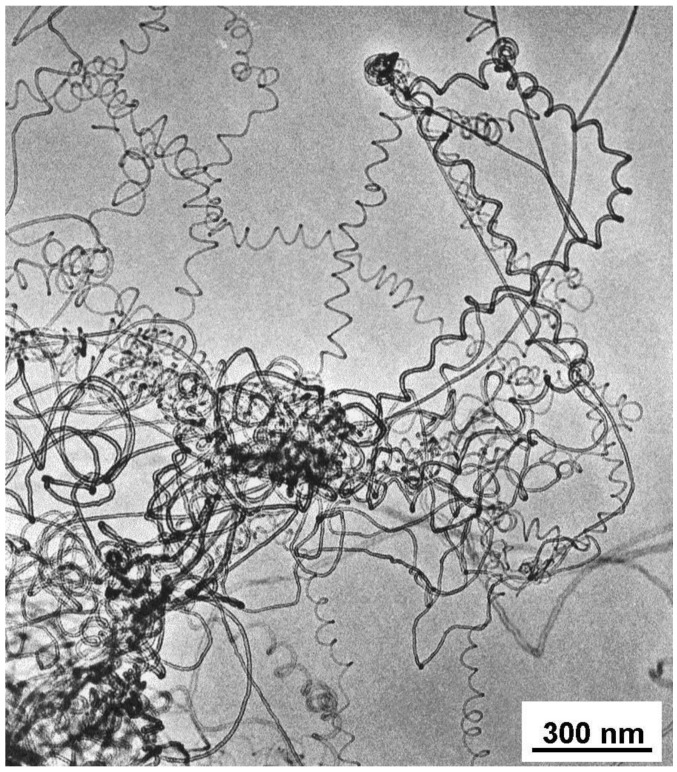
Carbon nanotubes formed in the decomposition of acetylene at 700 °C over Fe/silica (ion-adsorption precipitation using pH = 9 as initial solution, but the pH was close to 7 before filtration) catalyst. Reproduced from [77] with permission.

Further catalyst samples were made with iron by different preparation methods in order to improve both the quality and the quantity of as-prepared carbon nanotubes. The catalysts were tested in the decomposition of different hydrocarbons in the temperature range 650-800 °C using either fixed bed flow or fluidized bed reactor. The formation of coiled nanotubes was clearly enhanced in the catalyst formed using a pH = 9 initial solution (Figure 7). The existence of these helices has already been described [33,67,75,76] using supported Co/silica samples. Although Boehm [77] reported a frequent formation of coiled nanotubes on iron, most of the above-mentioned iron catalysts produced carbon nanotubes which were not regularly bent and thus no helices are present [78].

Cheng *et al*. synthesised helical carbon nanotubes by manganese nodule-catalyzed pyrolysis of acetylene. The synthesis conditions were similar to those reported earlier, except for the acetylene feed was 0-20 minutes delayed after the placement of the mineral catalyst at 750 °C. A triple helical carbon nanostructure was first observed in the product [79].

Mei Lu and his coworkers prepared coiled carbon nanotubes by catalytic chemical vapor deposition on finely dispersed cobalt nanoparticles supported on silicagel under reduced pressure and relatively low gas flow rates. The product was used in epoxy-based composites. TEM observations revealed different morphologies of carbon deposit, while HRTEM results indicated a graphitic lattice and polygonization in the sample. It was also found that the addition of few percentages of SWNTs could enhance the heat absorbability of the epoxy-based composite, while the incorporation of coiled carbon nanotubes in epoxy-based composites had a great influence on the heat shielding [80].

Wen and Shen synthesised coiled carbon nanofibers/nanotubes by Ni-catalyzed pyrolysis of acetylene, a small amount of the co-catalyst PCl_3_ was added into the acetylene gas. The role of the latter component was not convincingly clarified [81].

Highly compressed coiled carbon nanotubes can be prepared by spray pyrolysis of an ethanol solution containing cupric acetate as the catalyst precursor at a temperature of 850 °C. All coiled carbon nanotubes produced by Wang *et al*. possessed uniform shape with a sharp radius of curvature and a small coil pitch. The external diameter of the sample was about 30–50 nm with the inner diameter of about 10–20 nm. The growth of such coiled carbon nanotubes was probably related to the use of Cu as the catalyst. Copper containing catalyst particles may be in a melted or semimelted state under reaction conditions thus susceptible to the fluctuation of growth conditions induced by the periodical supply of the carbon source [82].

Varadan *et al*. used both thermal filament CVD and microwave CVD techniques for the fabrication of coiled carbon nanotubes. For catalysts preparation (Fe supported on magnesium carbonate, Fe supported on silica, Ni supported on zeolite), catalyst precursors were sprayed onto the graphite plate (for the thermal filament CVD furnace) or onto the SiC substrate (for the microwave CVD furnace) and then loaded into the reactors, the reaction temperature was at 700 °C. In the carbon deposit obtained hereafter, regularly coiled carbon nanotubes were present [83].

Carbon fibers were prepared by the catalytic decomposition of acetylene over finely dispersed cobalt, supported on amorphous silica, are often helix shaped. The stucture was studied by means of various electron diffraction techniques and by electron microscopy. It was shown that the tubules were hollow and consisted of concentric cylindar graphene sheets. In particular it was concluded that the helices are polygonized tubulus. It seemed evident that the catalytic particles played a role. It was alredy shown earlier that the catalytic particles were often found to be enclosed in the channel at the tip part of the fibres. Therefore it was assumed that during growth a catalytic particle was lifted by the growing fibre and that growth proceeded by the catalytic decomposition of the organic vapour in the contact region between particle and already deposited carbon [31].

As it was presumed earlier, the pH of the solution during catalyst preparation has a significant role. By treating the catalyst at different pH conditions, the shape of the metal particles was more irregular; during exothermic reaction of acetylene on the particle surface, the energy deposited by the reaction did not flow homogeneously through the whole particle and there could be areas with different catalytic activities. The assumption was that coil growth is rather similar to straight tube growth except for the fact that these different rates for the catalytic reaction at the surface of the particle probably induced different growth speeds around the catalyst particle. A higher carbon deposition rate at one side of the particle would generate the “outer” part of the spiral (following a spiral, a line of maximum length and a line of minimum length can be drawn). This is more likely to happen on bigger particles, which could also explain that the average diameter of the spirals is larger than that of the straight tubes.

**Figure 8:**
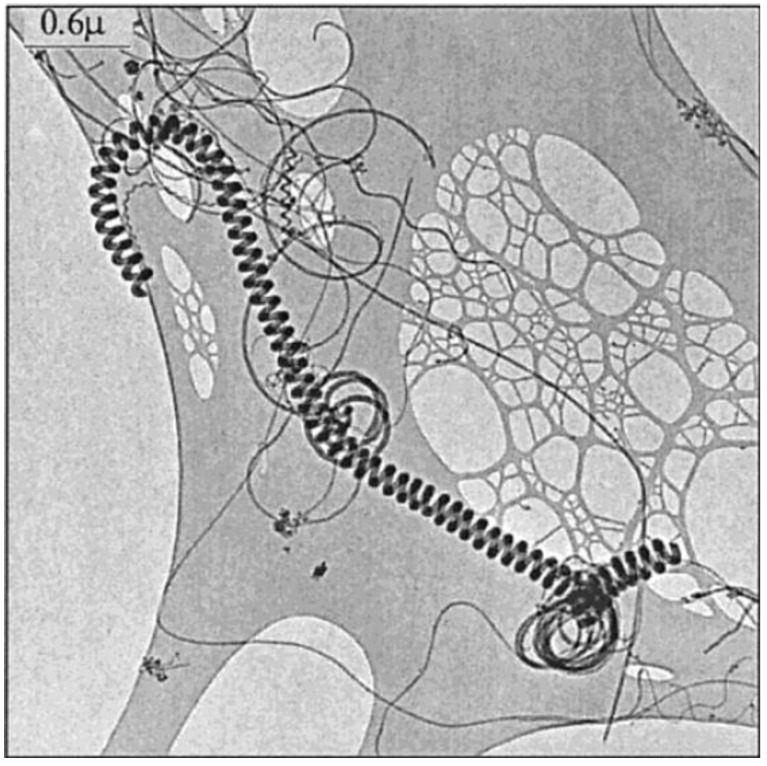
A typical coil, with its regular pitch. Reproduced from [25] with permission.

In order to prove above-mentioned hypothesis, catalyst series was prepared by precipitation of a cobalt acetate solution at different pH values. The catalyst used for this purpose was a cobalt catalyst supported on silicagel H. Cobalt acetate solution is a precursor which is well-suited to obtain proper catalyst particles. Decomposition of either cobalt acetate or cobalt hydroxide results in pure CoO on the surface upon heating. Since the solubility of cobalt salts depends strongly on the pH of the solution, the size and the shape of the deposited catalyst particles vary a lot with the pH of solution used during impregnation. During catalyst preparation the pH was set by ammonia addition so that the starting pH values could be measured for the different preparations: 7.5, 8, 8.5, 9, and 10. To each portion 1 g of catalyst support was added, then the preparation was filtered after 4 h. Catalytic nanotubes were grown by acetylene decomposition at 720 °C for 30 min with a gas feed of 70 L/h of nitrogen and 10 mL/min of acetylene. Figure 8 shows a coiled nanotube having a strikingly regular structure; with a pitch of 50 nm; the helix is regularly coiled like a nanometer size telephone cord.

The dimensions of the projections of the helices on a large number of tubes were measured to get their distribution for different pH values. This was facilitated by the fact that the measurements were done on TEM holey carbon grid samples, where the spirals lay flat.

**Figure 9:**
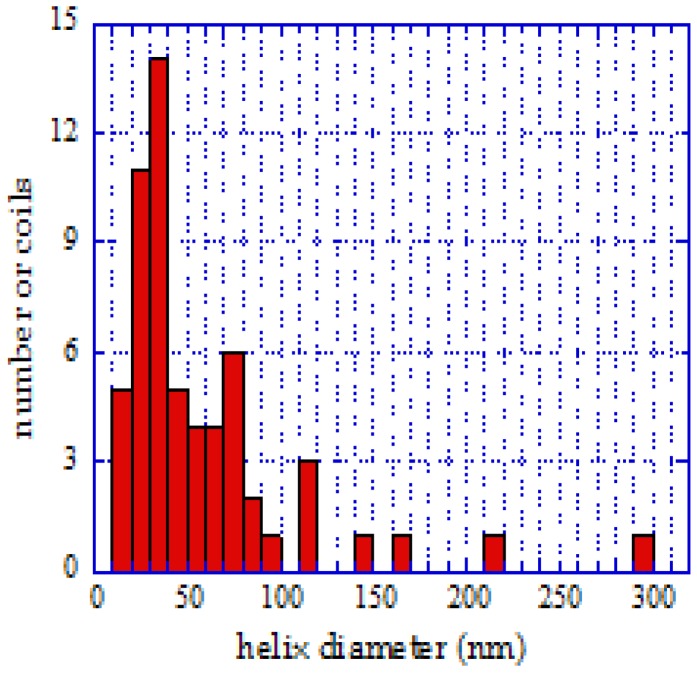

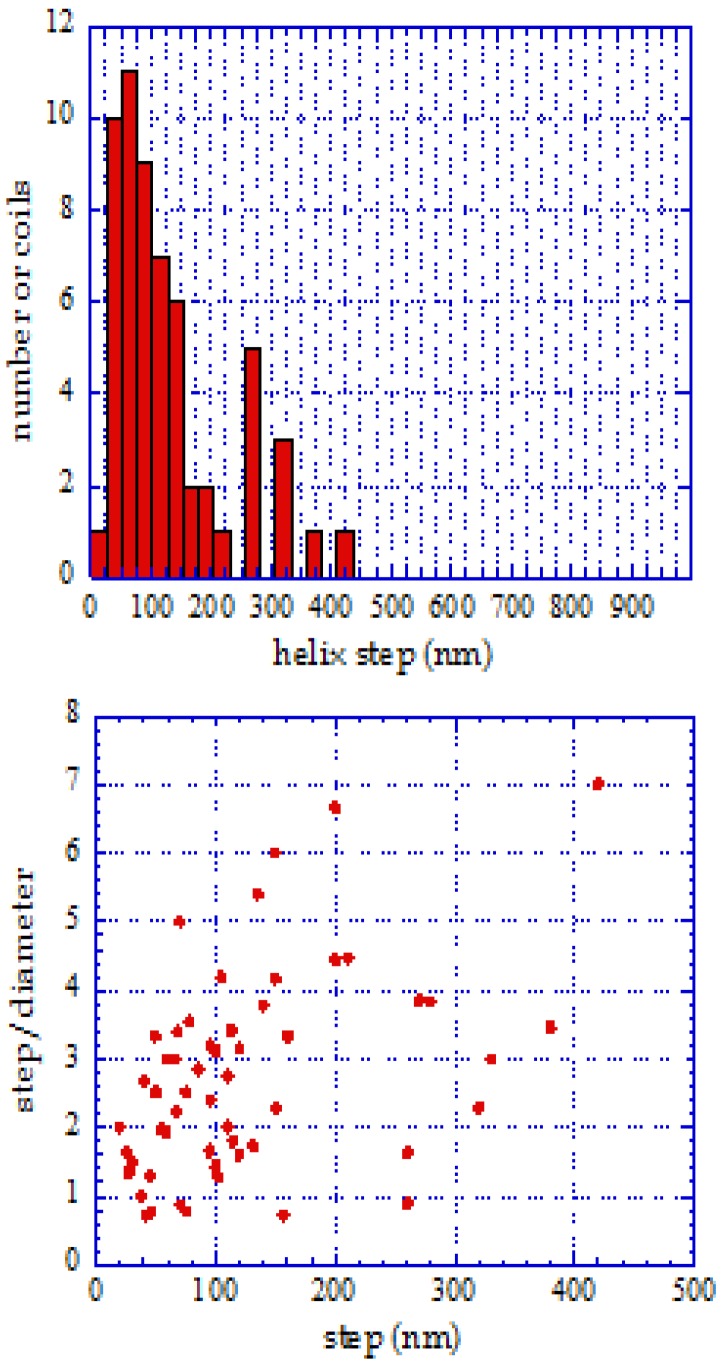
Distribution of diameters, helix pitch, and correlation between pitch and diameter at pH = 10. Reproduced from [25] with permission.

**Figure 10:**
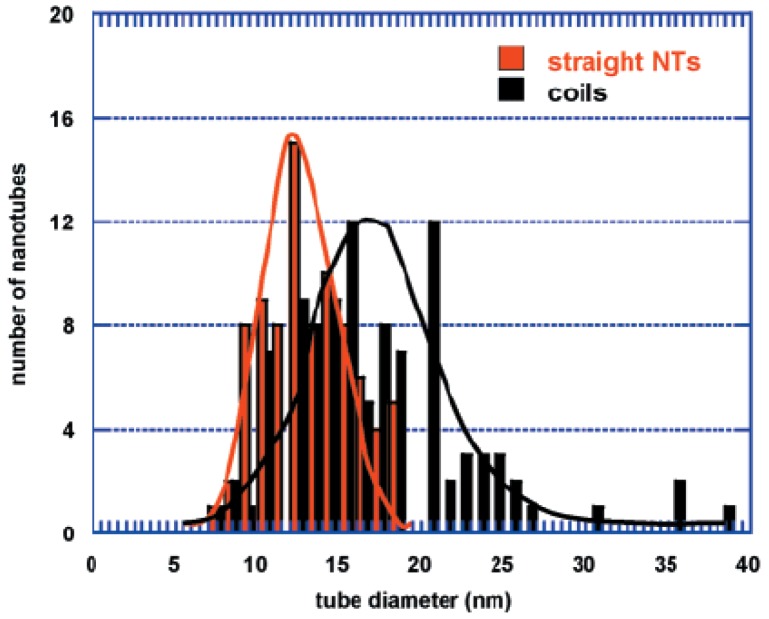
Comparison of tube diameter distributions for straight nanotubes and spirals. Reproduced from [25] with permission.

The geometrical parameters of the coils were already represented in Figure 1, where the pitch and tube diameter of a typical coil are shown. Samples of the size distributions at pH = 7.5 and pH = 10 were analyzed, and the diameter of these helical tubes was generally between 10 and 100 nm. The probability of deposition of larger and asymmetric particles increased with increasing pH. This observation is illustrated in Figure 9. The size distribution was wider than for the surrounding straight nanotubes, which was also clear from the statistics (Figure 10). The average diameter was larger as well (25 nm for the spirals on average, 15 nm for the straight tubes).

**Figure 11:**
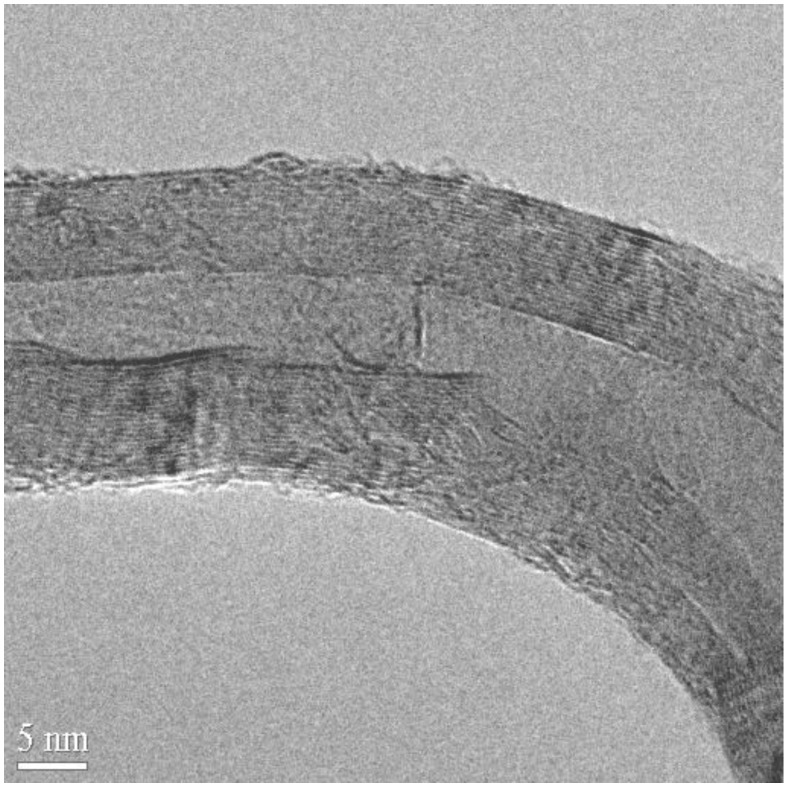
Defects allowing curvature at a strong bend of a spiral. On the upper part, the stretching of the planes leads to a regular curvature. In the inner part of the spiral both plane buckling, and interrupted layers close to the core can be seen. Reproduced from [25] with permission.

Characterization of the carbon deposits revealed that usually the spirals were real nanotubes, fairly well graphitized, with a core. Graphitization of the walls could be seen on the HREM micrographs, however, instead of polygonized structure presumed regularly in the past, irregular texture was observed very often (Figure 11). The less well-graphitized fibers were the thin ones with a pitch in the order of the diameter (tightly coiled helices), or the very large ones. The coils can have a great variety of diameters and pitches, appearing sometimes as slightly twisted tubes, at other times as tightly coiled spirals [25].

As a continuation of previous work, the influence of asymmetric catalytic particles prepared by various methods using the CVD method on the growth of spiral carbon nanotubes was investigated. Asymmetric particles (Fe, Co) were prepared by either milling or crystallization from oversaturated solution onto the surface of catalyst support or catalyst impregnation at pH 8-9. As-prepared catalysts were tested in the decomposition of acetylene at 720 °C. In order to increase the formation of coiled carbon nanotubes, some ball milled samples were treated in ammonia atmosphere, whereby the amount of spiral carbon nanotubes increased significantly [84].

## 5. Growth Mechanisms of Coiled Carbon Nanotubes

After the first experimental observation for the production of coiled carbon nanotubes Amelinckx *et al*. proposed the concept of a spatial-velocity hodograph to describe the extrusion of helix-shaped carbon nanotube from a catalytic particle quantitatively. In the same work they emphasized the importance of asymmetric catalytic particles in the formation of coiled carbon nanotubes. Due to local poisoning the catalytic activity of a particle may change with reaction time, consequently resulting carbon nanotubes may have various shapes such as a helix with a different pitch [20].

The pitch and diameter of a regular coiled carbon nanotube are basically determined by the length of the straight sections and by the distribution of bond shifts. As it was observed experimentally, the diameter and pitch of observed coiled tubules may differ to a great extent [18,19,20,25,31,32,85].

**Figure 12:**
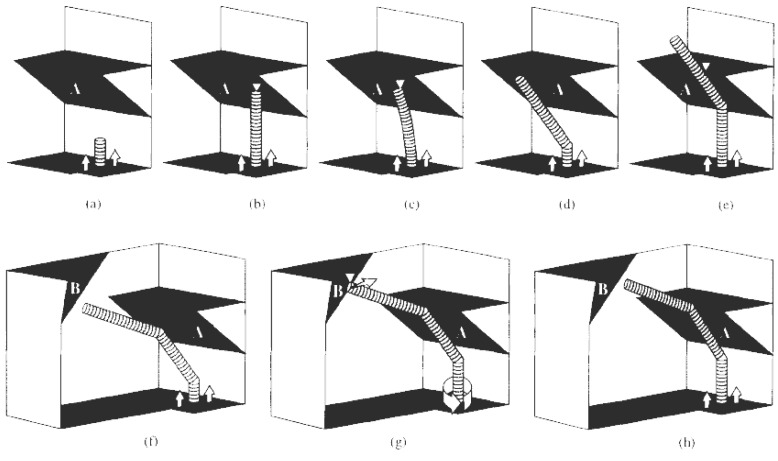
Explanation of the growth mechanism leading to tori (a)-(e) and to helices (a)-(h). (a) Growing nanotubule on an immobilized catalyst particle; (b) the tube reaches obstacle A; (c) elastic bending of the growing tubule caused by its blockage at the obstacle A; (d) after the formation of the knee, a second growing stage can occur; (e) second blockage of the growing nanotubule by the obstacle A; (f) after the formation of the second knee, a new growing stage can occur; (g) the tube reaches obstacle B; (h) formation of the regular helicity in the growing tubules by the obstacle B. Reproduced from [33] with permission.

A completely different approach was evolved in the steric hindrance model which was proposed to explain the possible formation of regular and tightly wound helices. If a growing straight tubule is blocked at its extremity, one way for growth to continue is by forming a knee close by the surface of the catalyst. The scheme of this hypothesis is sketched in Figure 12. Starting from the growing tubule represented in Figure 12(a), after blockage by *obstacle A* in Figure 12(b), first elastic bending can occur [Figure 12(c)]. Beyond a certain limit, a knee will appear close to the catalyst particle, relaxing the strain and freeing the tubule for further growth [Figure 12(d)]. If there is a single obstacle to tubule growth (*obstacle A* in Figure 12), the tubule will continue turning at regular intervals [Figure 12(e) and Figure 12(f)] but as it is impossible to complete a torus because of the presence of catalyst particle, this leads to the tightly wound helices already observed [67,86].

However, if there is a second obstacle to tubule growth [*obstacle B* in Figure 12(f)-(h)], forcing the tubule to rotate at the catalyst particle, the median planes of two successive knees will be different and the resulting tubule will be a regular helix. Note that the catalyst particle itself could act as the second *obstacle B*. The *obstacles A and B* of Figure 12 are hence considered as the bending driving forces, with *obstacle A* regulating the length of the straight segments and *obstacle B* controlling the rotation angle or number of rotational bond shifts [33].

In accordance with an early proposition concerning the role of asymmetric catalyst particles in the selective synthesis of coiled carbon nanotubes [20], further mechanistic investigations were done on this filed. From the point of view of catalyst preparation, it is interesting to consider what the processes which take place in the solution actually are. To form a Co precipitate, Co^2+^ can be either dissolved or precipitated as Co(OH)_2_ in the aqueous phase, and can be deposited on the surface of silicagel. With increasing NH_3_ concentration, amino-complexes of Co^2+^ have to be also taken into account. The following equilibria have an influence on ion concentrations in the solution during catalyst preparation:

Co^2+^ + 2 OH^–^ ↔ Co(OH)_2_

CH_3_COO^–^ + HOH ↔ CH_3_COOH + OH^–^

NH_3_ + HOH ↔ NH^4+^ + OH^–^

Co(NH_3_)^2+^_n_ NH_3_ ↔ Co(NH_3_)^2+^_n+1_     n = 0,1,2,…,5

Because the equilibrium constants for these steps are well-known, the Co distribution and actual pH could be calculated. Since silicagel support has a significant effect on both Co^2+^ concentration and pH, these calculations would be irrelevant for further considerations. Some kind of estimation can be given for determining free Co^2+^ by using the measured pH values and solubility product of Co(OH)_2_ (2.5 × 10^-16^). From these data it can be seen that the overwhelming part of Co^2+^ is precipitated. These particles which are colloidal in nature are likely to have an irregular shape. They can adsorb on the surface of the silicagel support. To vary the shape and the size of the cobalt clusters that precipitate, solutions of various pH were used for catalyst preparation. With increasing pH, Co(OH)_2_ aggregates are larger in size, and the formation of asymmetric adsorbates has a higher probability. This fact explains both the increasing number of helices and the higher average tube diameter obtained with increasing pH (Figure 9). Decreasing the yield with increasing pH can be explained by two facts: the formation of an amino-complex increases the solubility of Co at higher pH on one hand, and bigger catalyst particles result in smaller amount of active sites on the other hand.

It was already proved that, for straight tubes, the tube diameter depends on the average size of the catalyst particles [86]. To account for the other parameters that determine the shape of helices (diameter of the helix, pitch), the following considerations can be given. In many cases there is a strong interaction between the catalyst particles and the support. Indicating the importance of interaction, the activity of the catalyst strongly depends on the thickness of the CoO layer on the surface. If the catalyst particle deposited from a homogeneous solution is symmetric, the catalyst activity will be equal at the edge of this circular catalyst particle and acetylene decomposition will result in the formation of a straight carbon nanotube. When asymmetry appears in the spherical particle, helical tubes start to grow as a result of differences in local acetylene decomposition rate. If there is a big difference between maximum and minimum activity within the same particle, the curvature of the growing helical nanotube will be quite large, thus, the diameter of the helix will be large. With a lower activity difference, the diameter of the growing helix is smaller. Irregularities in the deposited catalyst particle can be significant. Consequently, a difference in activity has a crucial effect on the final shape of the tube [25].

## 6. Potencial Applications of Coiled Carbon Nanostructures

Theoretical studies and relevant characterizations predict excellent electrical, mechanical, and magnetic properties of regular coiled carbon nanotubes. Most likely their unique features derive from the combination of fascinating properties and specific helical morphology. Coiled carbon nanotubes will be of great interest probably not only in fundamental research but they will find interesting applications very soon either as nanoelectronic devices, or nanocomposites and nanoelectromechanical systems (NEMS). They are also considered as ideal materials for electromagnetic wave absorbents, tunable micro-devices, bioactivators, Li-battery electrodes and hydrogen containers, *etc.* [87,88,89].

In practical applications, helical carbon nanostructures have already been used in many fields such as fabrication of semiconducting infrared detection elements [90], flat panel field emission display [91], or microwave absorber [92]. Carbon nanocoils are also excellent candidates for future multipurpose innovations in nanodevices, specific sensors [93], nanovelcro, chiral catalysts, *etc.* [11].

Recently Volodin reported that it was possible to use coiled carbon nanotubes with attached electrodes as self-sensing mechanical resonators. After adsorption on a silicon substrate, coiled multiwalled nanotubes retained a 3D structure with sections of freely suspended windings. Some of these coiled carbon nanotubes produced by the CVD method had radii and pitches smaller than a few tens of nanometers. The resonance frequency of these tiny mechanical structures were well into the microwave GHz regime, making mechanics as fast as electronics. The self-sensing coiled nanotube sensors were well suited for measuring small forces and masses in the femtogram range [94]. Carbon nanocoils could be also suitable for helicoidal mass transport along them for example by a thermal gradient [95] or an electric field [96].

Recently, the development of carbon nanotubes opens a new alternative to reinforce the traditional composites. In particular, composites with metal matrix reinforced with carbon nanotubes are expected to have unique mechanical properties. However, using carbon nanotubes as reinforcing additives, either in metal- or polymer-based composites, two major obstacles still exist: the wettability of the carbon nanotube surface and the load transfer from the matrix to the nanotubes. To overcome these problems the coiled configuration of the nanotubes can be favourable. With helical structure the fracture toughness as well as mechanical strength of the composites can be improved significantly even if there is no direct chemical bonding between the coiled carbon nanotubes and matrix. Their coiled shape alone is favourable to induce mechanical [12]. It is clear that such carbon nanospirals would on one hand have a toughness resembling the toughness of nanotubes more than of carbon fibers, and that, on the other hand, if used in composites, they would be better anchored in their embedding matrix than straight nanotubes. Their shape would favor a better load transfer to the matrix than in the case of ordinary tubules, and possibly easier infiltration [25].

## 7. Conclusions

Various techniques have been developed during the last two decades for the synthesis of straight carbon nanotubes. Contrarily, selective production of regular coiled carbon nanotubes on a large scale is still a challenge. Investigations were mostly confined to theoretical calculations concerning their electrical properties. Attempts to measure the mechanical properties of coiled carbon nanotubes with the atomic force microscope have been made [94], but it is still not known what the spring constant of such a helix is.

Technological and industrial interest described in this review means an urging demand for speedy development on this field. It is hoped that further understanding of growth mechanism might help to control syntheses of carbon nanocoils by designing the features of the catalyst. With significant improvement of selective fabrication of asymmetric catalyst particles we might get closer to the solution of the problem of regularly coiled carbon nanotubes.
